# Liver X receptor-dependent inhibition of microglial nitric oxide synthase 2

**DOI:** 10.1186/s12974-015-0247-2

**Published:** 2015-02-10

**Authors:** Julie R Secor McVoy, Hanadi Ajam Oughli, Unsong Oh

**Affiliations:** Department of Neurology, Virginia Commonwealth University School of Medicine, PO Box 980599, VA 23298 Richmond, USA

**Keywords:** Liver X receptor, Microglia, Inflammation, HDAC, NF-kappaB, Central nervous system, Experimental allergic encephalomyelitis

## Abstract

**Background:**

The nuclear receptor liver X receptor (LXR) exerts transcriptional control over lipid metabolism and inflammatory response in cells of the myeloid lineage, suggesting that LXR may be a potential target in a number of chronic neuroinflammatory and neurodegenerative diseases where persistent microglial activation has been implicated in the pathogenesis.

**Methods:**

The effect of LXR activation on microglia and central nervous system (CNS) inflammation was studied using a synthetic LXR agonist in cultured microglia, a microglial cell line and experimental allergic encephalomyelitis (EAE), an animal model of CNS inflammation.

**Results:**

LXR activation inhibited nitric oxide synthase 2, inducible (*Nos2*) expression and nitric oxide production in lipopolysaccharide (LPS)-stimulated microglia. Inhibition of microglial activation in response to interferon-γ was less reliable. In LPS-stimulated cells, LXR activation did not inhibit nuclear translocation of NF-kappaB1 p50. Instead, LXR-dependent *Nos2* repression was associated with inhibition of histone 4 acetylation and inhibition of NF-kappaB1 p50 binding at the *Nos2* promoter. Histone acetylation and NF-kappaB1 p50 binding were mechanistically linked, and histone deacetylase (HDAC) activity appeared to be important for LXR-dependent transcriptional repression of *Nos2*. Analysis of CNS gene expression in animals undergoing EAE showed that the expressions of *Lxr* and LXR-dependent genes were downregulated during CNS inflammation. Nevertheless, administration of LXR agonist GW3965 during the effector phase of EAE delayed the onset of clinical disease and reversed the diminished expression of LXR-dependent reverse cholesterol transport genes. However, the CNS expressions of *Nos2* and other inflammatory genes were not significantly inhibited by LXR activation in EAE, and clinical disease severity was comparable to vehicle controls at later time points in LXR agonist treated animals.

**Conclusions:**

LXR can be targeted to modulate microglial activation. LXR-dependent repression of inflammatory genes may be stimulus-dependent and impaired by HDAC inhibition. Endogenous LXR activity does not appear to modulate CNS inflammation, but LXR activity can be partially restored in the CNS by administration of exogenous LXR agonist with an impact on clinical disease severity at early, but not late, time points in EAE.

## Background

Microglia are resident innate immune cells of the CNS and contribute to CNS inflammation and repair through multiple functions including scavenging, phagocytosis, antigen presentation and production of reactive oxygen/nitrogen species, cytokines and neurotropic factors [1]. Reactive microgliosis, or a state of persistent microglial activation, has been implicated in the pathogenesis of a number of neuroinflammatory and neurodegenerative diseases including multiple sclerosis, Parkinson disease and Alzheimer disease [2]. The production of excess reactive oxygen and reactive nitrogen species by activated microglia is thought to contribute to neurodegeneration by way of oxidative stress [3]. Inhibiting microglial activation or function has the potential to ameliorate neuroinflammatory and neurodegenerative disorders [4,5]. Defining the signals that control microglial activation, therefore, has important implications for modulating neuroinflammatory and neurodegenerative processes.

The liver X receptor (LXR) is an oxysterol-activated nuclear receptor [6]. Together with retinoid X receptor (RXR), with which LXR forms an obligate heterodimer, LXR controls gene transcription by modulating the function of nuclear receptor corepressor (NCoR) and nuclear receptor coactivator (NCoA) complexes [7]. LXR is known to control the expression of genes involved in reverse cholesterol transport such as *Abca1*, *Abcg1*, *Apoa1*, *Apoe* and *Cyp7a1* [8,9]. In cells of the myeloid lineage, LXR has also been shown to control inflammatory responses by transcriptional repression of a number of genes including *Nos2*, *Cox2* and *Il6* [10]. Thus, LXR is one among a number of biological signal pathways that link lipid metabolism and inflammation [11]. Previous studies have shown that activation of LXR by oxysterols inhibits proinflammatory responses in cultures of microglia and astrocytes, suggesting that the LXR pathway might serve a compensatory antiinflammatory function in response to oxidative stress [12,13]. In addition, previous studies have shown that LXR agonists reduced the severity of experimental allergic encephalomyelitis (EAE), an animal model of neuroinflammation, during its induction phase by an immunomodulatory effect on T helper lymphocyte differentiation [14,15].

Several questions remain regarding the role of LXR in CNS inflammation. It is unknown whether or not endogenous activation of LXR in the CNS functions to modulate the course of CNS inflammation. A related question is whether or not targeting LXR confers protection in the setting of already established CNS inflammation, independent of its immunomodulatory effects on peripheral lymphocytes. We examined LXR function and the mechanism of transcriptional repression in cultured microglia as well as the effect of LXR activation during CNS inflammation.

## Methods

### Reagents

Culture media, fetal calf serum, all media supplements, buffered solutions, Griess reagent kit and RNAi Max were from Life Technologies (Carlsbad, CA). GW3965 and fluorobexarotene were from R & D Systems (Minneapolis, MN). LPS, trichostatin A, C646 and TMB peroxidase substrate were from Sigma-Aldrich (St. Louis, MO).

### Primary murine microglial cultures

Timed-pregnant ICR mice were purchased form Harlan (Indianapolis, IN). Primary microglia-enriched cultures were prepared from whole brains of 1- to 2-day-old ICR pups using a previously described protocol [16]. Briefly, following removal of meninges and blood vessels, brains were mechanically dissociated by trituration then seeded in 150-cm^3^ flasks in supplemented DMEM/F12 media containing 10% fetal calf serum (4 to 5 brains per flask). Media were replaced at day 3 and 7 of culture. Microglia were shaken off at days 14 and 21 of culture and re-plated at 1 × 10^5^ cells/well in DMEM containing 2% fetal calf serum. Cells were treated 24 h after re-plating.

### Real-time reverse transcription-polymerase chain reaction (RT-PCR)

Real-time RT-PCR analyses of *Abca1*, *Abcg1*, *Apoa1*, *Nos2*, *Il1b*, *Il6*, *Lxra*, *Lxrb* and *Tnf* were performed using commercially available primer and probes sets and Taqman RNA-to-C_T_ 1-Step Kit (Life Technologies, Carlsbad, CA) according to the manufacturer’s protocol. Total RNA was extracted using RNeasy Mini Kit (Qiagen, Valencia, CA) according to the manufacturer’s protocol. Relative quantitation of mRNA was performed using the comparative threshold (delta delta C_T_) method [17] using *Actb* as endogenous control.

### Cytokine ELISA

Interleukin-1β (IL-1β) and tumor necrosis factor (TNF) in conditioned media were measured using the antibodies and reference standards contained in R & D Systems DuoSet ELISA kits (R & D, Minneapolis, MN) according to the manufacturer’s protocol. Test samples and reference standards were incubated for 2 h in wells that had been coated overnight with 0.8 μg/ml goat anti-mouse TNF or 4.0 μg/ml rat anti-mouse IL-1β capture antibodies in phosphate-buffered saline (PBS) and blocked for 1 h with 300 μl of 1% w/v bovine serum albumin (BSA) in PBS. Captured cytokines were detected with biotinylated goat anti-mouse TNF or IL-1β detection antibodies, visualized by incubation for 30 min with streptavidin-HRP followed by timed incubation with TMB peroxidase substrate. The enzyme reactions were quenched by addition of 2 N H_2_SO_4,_ and the absorbance of the resulting solutions was measured using a microplate spectrophotometer (BioTek, Winnoski, VT). The cytokine concentration in each test sample was calculated by the instrument software using the formula of the four-parameter best-fit curve to the standards.

### Griess assay (nitrite production)

Fresh or frozen aliquots of conditioned media were analyzed for the presence of nitrite using the reagents provided in the Griess Reagent Kit according to the manufacturer’s protocol. The reaction was started by the addition of 20 μl of freshly prepared Greiss reagent to wells containing 200 μl of deionized water and 50 μl of sample media or standards (1–100 μM sodium nitrite prepared in naïve media). Following a 30-min incubation at room temperature in the dark, the absorbance at 548 nm was measured using a microplate spectrophotometer.

### Immunoblotting

Nuclear extracts of BV2 cells were isolated using the NE-PER nuclear and cytoplasmic extraction reagent (Thermo Scientific, Rockford, IL) according to the manufacturer’s protocol. Protein concentration was measured using DC Protein Assay (Bio-Rad, Hercules, CA). Nuclear extracts were combined with a Laemmli sample buffer (Bio-Rad) separated on 10% Mini-PROTEAN TGX precast gels (Bio-Rad) at 200 V for 35 to 40 min, then transferred to a polyvinylidene difluoride membrane (Bio-Rad) at 30 V over 24 h at 4°C. Following transfer, the membrane was blocked with PBS/10% Tween-20/5% dry milk for 30 min, then incubated overnight with anti-p50 antibody (sc-1190, Santa Cruz Biotechnology, Dallas, TX) diluted 1:500 in PBS/10% Tween-20/5% dry milk at 4 C. After three washes in PBS/10% Tween-20, the membrane was incubated with donkey anti-goat IgG antibody conjugated to HRP (sc-2020, Santa Cruz Biotechnology) diluted 1:10,000 in PBS/10% Tween-20/5% dry milk for 45 min at room temperature. After 3 washes in PBS, the bands were visualized using SuperSignal West Femto Chemiluminescent Substrate (Thermo Scientific). Chemiluminescence was detected and saved as TIFF file images using a digital imaging system (VersaDoc MP 4000, Bio-Rad). For beta-actin immunoblot, membranes were stripped using Restore Western Blot Stripping Buffer (Thermo Scientific), then incubated in anti-beta actin antibody (clone AC-74, Sigma Aldrich) diluted 1:10,000 in PBS/10% Tween-20 for 3 h at room temperature, followed by washes and incubation in donkey anti-mouse IgG antibody conjugated to HRP (sc-2314, Santa Cruz Biotechnology) diluted 1:10,000 in PBS/10% Tween-20. Beta-actin bands were visualized using Immobilon Western Chemiluminescent HRP Substrate (EMD Millipore, Billerica, MA). Chemiluminescence images were analyzed on ImageJ to acquire relative density measurements. p50 band relative density measurements were normalized to corresponding beta-actin relative density measurements for each sample.

### Chromatin immunoprecipitation (ChIP)

ChIP assays were performed using the Magna ChIP A/G kit (Millipore, Billerica, MA) according to the manufacturer’s protocol. BV2 cells were plated in 15-cm culture dishes overnight. Following treatment, cells were fixed in freshly prepared formaldehyde (1% final concentration) to cross-link protein and DNA. Cells were scraped and re-suspended in protease inhibitor cocktail containing buffer, lysed, then sheared by sonication. Genomic DNA fragments were typically less than 500 bases in size; 10-μl (2%) aliquots were set aside as input. Following overnight immunoprecipitation with anti-acetylated histone 4 antibody (Millipore, Billerica, MA) or anti-NF-kappaB1 p50 antibody (sc-1190, Santa Cruz Biotechnology, Dallas, TX), protein/DNA cross-linking was reversed and DNA purified prior to assay by PCR. Primers for PCR were designed using Primer3 software [18] based on *Nos2* gene and 5′ flanking sequences obtained from UCSC Genome Bioinformatics [19]. Primer sequences are listed in Table 1. PCR was performed using QuantiFast SYBR Green PCR Kit (Qiagen, Valencia, CA) on a real-time PCR instrument (Life Technologies, Carlsbad, CA). Percent input was calculated as the delta C_T_ between input DNA and ChIP DNA. Fold enrichment was calculated using the delta delta C_T_ method with normalization against either isotype IgG ChIP DNA or a negative locus primer set.

**Table 1:** **PCR primer sequences**

**Primer ID**	**Forward**	**Reverse**	**Position relative to TSS**
Nos2_ChIP	GGAGTGTCCATCATGAATGAG	CAACTCCCTGTAAAGTTGTGACC	−159 to −15
Nos2_ChIP2	CCAGAACAAAATCCCTCAGC	CTCATGCAAGGCCATCTCTT	−355 to −198
Nos2_ChIP3	AGCACAGCCCATCCACTATT	CGGAGCTCTCTGGCTTTCT	−551 to −359
Nos2_ChIP4	AAAGGCTTATGCCACCACAC	TTTTCTCAGTGGCATTCTCTCTC	−748 to −572
Nos2_ChIP5	AATTCCATGCCATGTGTGAA	TGATCCCTGAGTTTGGGCTA	−936 to −779
Nos2_ChIP6	TTGAGGCCACACACTTTTTG	TGACAGTGTTAGGGGAAAAGG	−1124 to −959
Nos2_ChIP7	TCACCACACCCAGCATTTTA	GGGAGATGGCTCAGTTGGTA	−1387 to −1188
Nos2_ChIP8	GGGTGTTGCCTGGATAAAGA	GTTCCAGGCTGGTGAGAGAT	−1654 to −1472
Nos2_ChIP9	AAGGGTTCCATTGTGACAAAC	CCCAATACTTGGGAAAGACG	−1855 to −1663
Nos2_ChIP10	CAGCCAAGCACTCCAATGTA	TTACAGCTACGCCTGCAACA	−2002 to −1880
Nos2_ChIP11	AAACTTCTCAGCCACCTTGG	TTCCCAAGCAGGAAGACACT	30 to 189
Nos2_ChIP12	AAACCAGGCTTTCCCTTCTC	TTGCAGAGAAGAAATCTGACCA	198 to 336
Nos2 a	GCTTCACTCAGCACAGCCCATCCAC	GCTAAACCACATACCCTGGCTTGCAG	−560 to −308
Nos2 b	GCCAGCCTCCCTCCCTAGTGAGTCC	GACCCTGGCAGCAGCCATCAGGTAT	−299 to −34
Nos2 c	CCTGCTTGGGAAAGCCCAGAAAACC	TGGGGCAGAGGGCACATCTCATAAA	178 to 462
TNF a	TCTAAATGGGACATCCATGGGGGAGA	TCCGTGAATTCCCAGGGCTGAGTTC	−673 to −424
TNF b	ACACCCTCCTGATTGGCCCCAGATT	CTTGCTGTCCTCGCTGAGGGAGCTT	−278 to 18
TNF c	CCCCTCCACACTCTCCTCCACCTTG	GGCAGAAGAGGCACTCCCCCAAAAG	191 to 464
GAPDH	AGTGCCCACTCCCCTTCCCAGTTTC	GAGGCCCAGCTACTCGCGGCTTTA	−178 to 98
Rhodopsin	CAGCCTGAGGCCACCAGACTGACAT	CACAGCGCAACTCCAGGCACTGAC	−268 to −6

### DNase accessibility assay

DNase accessibility was measured in primary microglia cells using the EpiQ Chromatin Analysis Kit (BioRad, Hercules, CA) according to the manufacturer’s protocol. Microglia were plated in 48-well culture plates at a density of 2 × 10^5^ cells per well and incubated overnight prior to treatment. Following treatment, chromatin was digested *in situ* by incubation at 37°C for 15 min with 100 μl of EpiQ chromatin buffer containing 2 μl DNase. Cells incubated with chromatin buffer without added DNase were used as the undigested control. Addition of Stop Buffer halted DNase activity and caused cells to detach during a 10-min incubation at 37°C. The resulting cell solutions were combined with Lysis Solution and 100% ethanol, mixing after each addition, and then transferred to the supplied mini columns for isolation of DNA. Following the application of alternating low and high stringency wash solutions, purified genomic DNA was eluted using DNA elution solution preheated to 70°C. Samples were analyzed by quantitative PCR using primers designed to amplify *Nos2* and *Tnf* genes, promoters and flanking regions. Primers were designed using Primer3 software [18] based on sequences obtained from UCSC Genome Bioinformatics [19]. Primer sequences are listed in Table 1. Delta C_T_ was calculated as the difference in C_T_ between undigested and DNase digested samples. Low absolute value delta C_T_ corresponded to genomic DNA with high nucleosome occupancy (heterochromatin), and high absolute value delta C_T_ corresponded to genomic DNA with low nucleosome occupancy (euchromatin). The promoter region of the rhodopsin (*Rho*) gene, which is not expressed in microglia, consistently showed no shift in C_T_ between the undigested and digested samples, indicating a heterochromatin structure. Conversely, the promoter region of *Gapdh*, which is constitutively expressed, consistently showed a shift in C_T_ between the undigested and digested samples, indicating an euchromatin structure (data not shown). *Percent chromatin accessibility* was calculated using the delta delta C_T_ method using *Rho* gene promoter as the reference region: (1- (2^(delta C_T_ target gene - delta C_T_*Rho*))) × 100.

### Transfection with small interfering RNA (siRNA)

Primary murine microglia were transfected with siRNA directed at HDAC3 or non-targeting siRNA (10 pmol/1 × 10^5^ cells; Dharmacon) using RNAi Max according to the manufacturer’s protocol; 72 h following transfection, cells were treated, and RNA was obtained using the RNeasy Mini Kit (Qiagen). The efficiency of RNA knockdown was routinely 70–80% based on real-time RT-PCR.

### EAE: induction and treatment

EAE was actively induced in 6 to 8-week-old (17 to 20 g) C57BL/6 mice purchased from Harlan (Indianapolis, IN) as previously described [20]. Mice were administered subcutaneous injection of 300 μg myelin oligodendrocyte glycoprotein peptide (MOG_35–55_) in complete Freund’s adjuvant, followed by intraperitoneal injection of 300 ng pertussis toxin. Pertussis toxin injection was repeated 48 h later. A control group of mice received complete Freund’s adjuvant and pertussis toxin without MOG_35–55_. Groups of mice were administered daily intraperitoneal injections of either GW3965 or vehicle beginning day 8 post induction. GW3965 was dissolved in dimethyl sulfoxide (DMSO), mixed 1 to 1 with PBS, and 50 μl of this mixture containing 500 μg of GW3965 (or 25 to 30 mg/kg) was administered daily. Vehicle controls received 50 μl mixture of DMSO and PBS (1:1) administered daily. Mice were scored as follows: 0, no overt signs of disease; 1, limp tail or hind limb weakness but not both; 2, limp tail and hind limb weakness; 3, partial hind limb paralysis; 4, complete hind limb paralysis; 5, moribund state. Primary clinical outcome was the mean cumulative EAE score (sum of clinical scores from induction until the day of sacrifice for individual mice divided by the number of mice). Mice that did not get sick were excluded from analysis. Following euthanasia, animals underwent transcardiac perfusion with up to 50 ml of normal saline using a rate-controlled pump. Spines were dissected and cords were pushed out of the column using hydraulic pressure applied through a 19-gauge needle and syringe filled with PBS.

### Fluorescence-assisted cell-sorting (FACS) analysis

Following euthanasia, the cervical and axillary lymph nodes and spleen were removed. Single cell suspensions were prepared by grinding the tissue over a 70-μm sterile cell strainer using the rubber end of a syringe plunger then washing with RPMI media. Cells were washed again and re-suspended in RPMI supplemented with L-glutamine, penicillin/streptomycin and 10% FCS. Cells were then plated in 24-well plates and stimulated with PMA/ionomycin/brefeldin A for 6 h as previously described [21]. Cells were fixed with 4% paraformaldehyde, permeabilized with Perm/Wash Buffer (BD Biosciences, San Jose, CA), then incubated with anti-CD3-PE-Cy5 (clone 17A2), anti-CD4-FITC antibody (clone GK1.5), anti-interleukin-17A-PE antibody (clone TC11-18H10) and anti-interferon-γ-APC antibody (clone XMG1.2, BD Biosciences) for 30 min. FACS data were acquired on a FACSCalibur (BD Biosciences) and analyzed using FlowJo software (FLOWJO, Ashland, OR). FSC vs. SSC plots were used to gate on live cells. CD3 vs. CD4 plots were used to gate on CD3 + CD4+ cells. Interleukin-17 vs. interferon-γ plots were used to gate on interleukin-17 positive, interferon-γ positive and double-positive cells.

### Statistical analysis

Mann–Whitney was used for two group comparisons. Kruskal-Wallis test with Dunn’s post-hoc analysis was used for multiple comparisons. Statistical analysis was performed using Prism software (GraphPad, La Jolla, CA).

## Results

### LXR inhibits reactive nitrogen species production in cultured microglia

**Figure 1:**
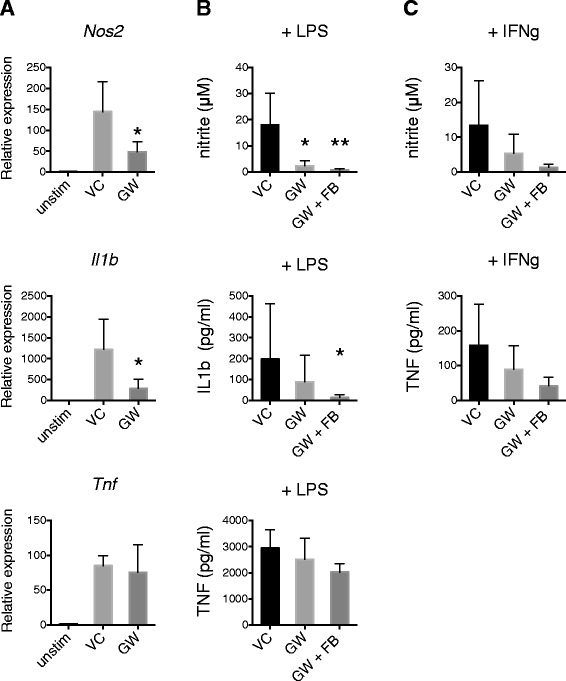
**LXR inhibits reactive nitrogen species production in cultured microglia.** Primary murine microglia were pretreated with the LXR agonist GW3965 (5 μM) with or without RXR agonist fluorobexarotene (0.1 μM) for 1 h then stimulated with LPS (10 ng/ml) or interferon-γ (10 ng/ml) for 48 h. Real-time RT-PCR was used to measure RNA expression (relative expression). Nitrite was measured by Griess assay as an indicator of nitric oxide production. IL-1β and TNF were measured by ELISA. **A** The effect of GW3965 (GW) or vehicle (VC, 0.05% DMSO) on LPS-induced RNA expression of *Nos2*, *IL1b* and *Tnf* compared to unstimulated (*unstim*) cells. **B** The effect of GW3965 (GW) with or without fluorobexarotene (FB) on LPS-induced nitrite, IL-1β and TNF production/release. **C** The effect of GW3965 (GW) with or without fluorobexarotene (FB) on interferon-γ-induced nitrite and TNF production/release. Data are mean + SD. *N* = 6 independent experiments, except column A (*N* = 5). Kruskal-Wallis test: *< 0.05, **< 0.001.

The synthetic LXR agonist GW3965 was used to study the effect of LXR activation in primary cultures of murine microglia. Stimulation with LPS resulted in activation of microglia as evidenced by increased nitric oxide, IL-1β and TNF production. The addition of GW3965 to culture resulted in reduced *Nos2* transcript and significant reduction in LPS-induced nitric oxide (nitrite) production/release (Figure 1A and B). The presence of RXR agonist fluorobexarotene potentiated the inhibitory effect of LXR agonist on LPS-induced nitric oxide production/release. LXR activation also had a consistent partial inhibitory effect on LPS-induced IL-1β transcriptional activity and production/release, which was potentiated by the presence of RXR agonist. The effect of LXR agonist on interferon-γ-induced *Nos2* activation was less reliable (Figure 1C). Furthermore, we did not observe a reliable effect of LXR activation on microglial production of TNF.

### LXR activation is associated with histone deacetylation and inhibition of p50 binding at the Nos2 promoter

**Figure 2:**
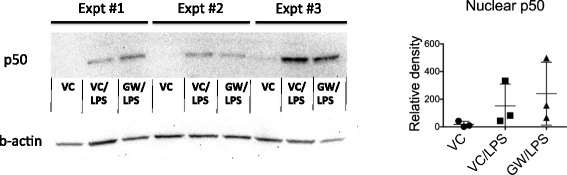
**LXR activation does not inhibit nuclear translocation of NF-kappaB1 p50.** Nuclear extracts from microglial cell line BV2 were isolated following 3 h LPS (10 ng/ml) stimulation. Cells were pretreated with either LXR agonist GW3965 (5 μM) or vehicle (0.05% DMSO). NF-kappaB1 p50 was detected by immunoblotting. Relative density measurements of the p50 bands were normalized to relative beta-actin density. *Left figure* shows p50 and corresponding beta-actin immunoblots for unstimulated (VC), vehicle pretreated/LPS stimulated (VC/LPS) and GW3965 pretreated/LPS stimulated (GW/LPS) samples from three independent experiments (“Expt”). *Right figure* shows a summary plot of the normalized relative density measurements of the p50 bands for each condition.

To investigate the mechanism of LXR-dependent inhibition of LPS-induced microglial activation, the effect of LXR activation NF-kappaB signaling was examined. Nuclear extracts were isolated from microglial cell line BV2 to detect nuclear translocation of NF-kappaB1 p50 by immunoblotting. Figure 2 shows that the levels of nuclear p50 are increased following LPS stimulation, indicating nuclear translocation of NF-kappaB1 p50. Pretreatment with the LXR agonist GW3965 did not reduce the levels of nuclear p50 in the LPS-stimulated cells. The results indicate that LXR activation does not inhibit nuclear translocation of NF-kappB1 or otherwise reduce the levels of nuclear NF-kappaB1.

**Figure 3:**
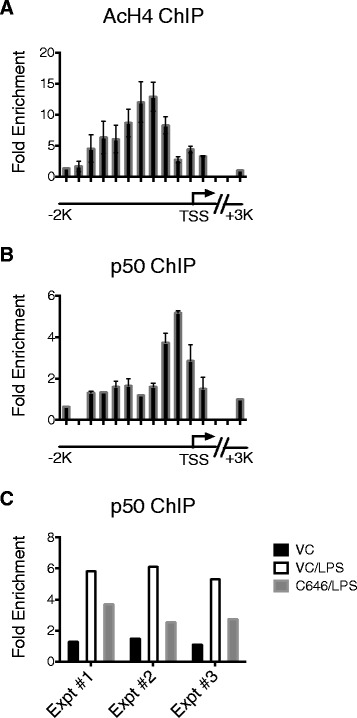
**Stimulation of microglia results in histone acetylation and NF-kappaB1 p50 binding at the Nos2 promoter** ***.*** Chromatin immunoprecipitation (*ChIP*) was performed on microglial cell line BV2 following 3 h LPS (10 ng/ml) stimulation with or without histone acetyltransferase inhibitor C646. **A** Acetylated histone 4 (AcH4) ChIP on LPS-stimulated BV2. AcH4 ChIP using primers spanning 2 kb upstream of *Nos2* transcriptional start site (TSS) showed that the peak of histone 4 acetylation is detected by primers amplifying −551 to −359 bases upstream of *Nos2*. Mean + SD. *N* = 3. **B** NF-kappaB1 p50 ChIP on LPS-stimulated BV2. p50 ChIP using primers spanning 2 kb upstream of *Nos2* TSS demonstrated the peak of p50 binding at −159 to −15 bases upstream of *Nos2*. Mean + SD. *N* = 3. **C** The effect of pretreatment with histone acetyltransferase inhibitor C646 (*C646/LPS*) on NF-kappaB1 p50 binding detected by ChIP at the *Nos2* promoter compared to unstimulated (*VC*) and vehicle treated (*VC/LPS*) cells. Data shown for primers amplifying −159 to −15 bases upstream of *Nos2* TSS: three independent experiments (Expt).

Previous studies reported that LXR associates with histone deacetylase (HDAC) 3 via the LXR-NCoR complex, suggesting that the transcriptional repression by the LXR-NCoR complex involves histone modification [22]. To examine the effect of LXR activation on histone modification at the *Nos2* promoter and its impact on LPS-induced NF-kappaB signaling [23], ChIP was performed on microglial cell line BV2 using antibodies specific to acetylated histone 4 and NF-kappaB1 p50. Figure 3 shows results from the promoter “tiling” ChIP experiment using primers that collectively amplify a ~2 kilobase region upstream of the *Nos2* gene. Following LPS stimulation of BV2 cells, a peak of histone 4 acetylation was detected at −551 to −359 bases upstream of the *Nos2* transcriptional start site (Figure 3A). The peak of NF-kappaB1 p50 binding was detected at −159 to −15 bases upstream of the *Nos2* transcriptional start site upon LPS stimulation (Figure 3B). To investigate the relationship between histone acetylation and NF-kappaB1 p50 binding at the *Nos2* promoter, cells were pretreated with C646, an inhibitor of histone acetyltransferase [24]. Inhibition of histone acetyltransferase resulted in reduced NF-kappaB1 p50 binding at the *Nos2* promoter, suggesting that histone acetylation functions to regulate NF-kappaB1 p50 occupancy at the *Nos2* promoter (Figure 3C).

**Figure 4:**
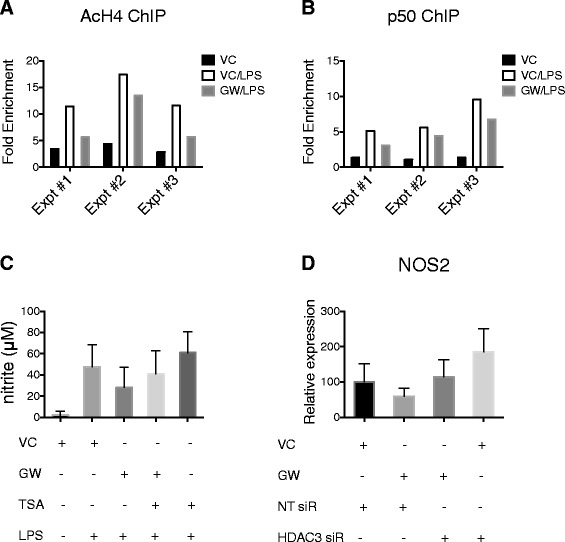
**LXR activation is associated with histone deacetylation and inhibition of p50 binding at the Nos2 promoter. A** AcH4 ChIP at the *Nos2* promoter for LPS-stimulated BV2 cells pretreated with 5 μM GW3965 (*GW/LPS*) compared to unstimulated (*VC*) and vehicle pretreated (*VC/LPS*) cells. Data shown for primers amplifying −551 to −359 upstream of *Nos2*. **B** NF-kappaB1 p50 ChIP at the *Nos2* promoter for LPS stimulated BV2 cells pretreated with 5 μM GW3965 (*GW/LPS*) compared to unstimulated (*VC*) and vehicle pretreated (*VC/LPS*) cells. Data shown for primers amplifying −159 to −15 bases upstream of *Nos2*. **C** Microglial cell line BV2 were pretreated with 1 μM GW3965 (*GW*), HDAC inhibitor trichostatin A (*TSA*, 10 nM) or vehicle (*VC*, 0.1% DMSO) for 1 h, then stimulated with LPS (10 ng/ml). Nitric oxide (nitrite) production was measured by Griess assay. Data are mean + SD. *N* = 4 independent experiments. **D** Primary murine microglia were transfected with HDAC3 siRNA (*HDAC3 siR*) or non-targeting siRNA (*NT siR*); 72 h following transfection, cells were pretreated with 1 μM GW3965 (*GW*) or vehicle (*VC*, 0.05% DMSO) for 1 h followed by stimulation with LPS (10 ng/ml). *Nos2* gene expression (relative expression) was determined by real-time RT-PCR. Data are mean + SD. *N* = 3 independent experiments.

LXR activation inhibited both histone acetylation and NF-kappaB1 p50 binding at the *Nos2* promoter. Pretreatment of BV2 cells with GW3965 resulted in reduced histone 4 acetylation (Figure 4A), suggesting the action of HDAC on the *Nos2* promoter following LXR activation. Furthermore, pretreatment with LXR agonist GW3965 resulted in a reduction of p50 binding at the *Nos2* promoter (Figure 4B), indicating LXR-dependent inhibition of NF-kappaB signaling. To test the hypothesis that the LXR-dependent repression of *Nos2* requires HDAC activity, BV2 cells were cultured with or without HDAC inhibitor trichostatin A. Pretreatment with trichostatin A partially reversed the inhibitory effect of GW3965 on nitrite production (Figure 4C), suggesting that the LXR-dependent inhibition of nitric oxide (nitrite) production was mediated in part by the action of HDAC. The addition of TSA alone increased nitrite production above baseline, suggesting that HDAC activity maintains basal repression of nitric oxide production. Similar results were observed following HDAC3 knockdown (Figure 4D). Transfection with siRNA targeting HDAC3 partially reversed the inhibitory effect of GW3965 on *Nos2* gene expression. Transfection with HDAC3 siRNA alone increased *Nos2* gene expression above baseline, indicating that HDAC3 activity maintains basal transcriptional repression of *Nos2*.

### DNase accessibility assay defines an inducible nucleosome depleted region in the Nos2 promoter, but DNase accessibility is not altered by LXR activation

**Figure 5:**
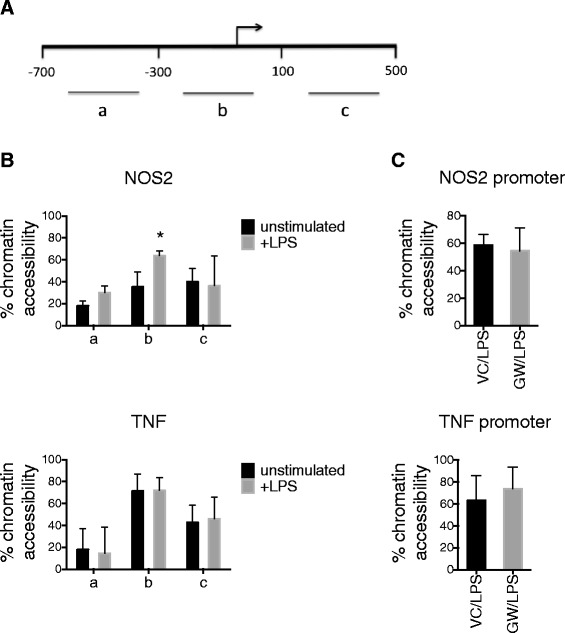
**DNase accessibility assay defines an inducible nucleosome-depleted region on the Nos2 promoter, but DNase accessibility is not altered by LXR activation.** Cultured microglia were subjected to *in situ* chromatin digestion by DNase, followed by quantitative PCR. The shift in C_T_ values between digested and undigested chromatin reflected nucleosome occupancy. DNase accessibility (% chromatin accessibility) was quantified using the delta delta C_T_ calculation, normalizing to the *Rho* promoter. Low % chromatin accessibility indicated high nucleosome occupancy and a high % chromatin accessibility indicated nucleosome depletion. **A** Schematic representation of genomic DNA regions amplified following DNase digestion relative to transcription start site (*right-angle arrow*). **B** The proximal 5′ region flanking the *Nos2* gene (b) showed low accessibility in the unstimulated state and significantly higher DNase accessibility following LPS stimulation. The proximal 5′ region flanking the *Tnf* gene (b) showed constitutive high DNase accessibility. Data are mean + SD. *N* = 4 independent experiments. *< 0.05, Mann–Whitney. **C** DNase accessibility is not altered at the *Nos2* promoter in the presence of LXR agonist GW3965 (5 μM, GW/LPS). Data for *Tnf* promoter are also shown. Data are mean + SD. *N* = 4 independent experiments.

We next sought to determine the scale of epigenetic modification involved in LXR-dependent *Nos2* repression. Specifically, we asked whether or not LXR-dependent repression involves alteration in nucleosome occupancy over the *Nos2* promoter. We used *in situ* DNase digestion followed by quantitative PCR to identify regions of genomic DNA with high nucleosome occupancy or heterochromatin and regions of genomic DNA with low nucleosome occupancy or euchromatin. In unstimulated primary murine microglia, the proximal 5′ flanking region of the *Nos2* gene showed low DNase accessibility, indicating heterochromatin structure (Figure 5B). Upon LPS stimulation, however, DNase accessibility of the *Nos2* promoter region increased significantly, indicating inducible nucleosome depletion and transition to euchromatin structure. In contrast, the proximal 5′ flanking region of the *Tnf* gene showed high DNase accessibility in both the unstimulated and LPS-stimulated states, indicating constitutive euchromatin structure of the *Tnf* promoter (Figure 5B). To test whether or not LXR-dependent *Nos2* repression involves alteration in nucleosome occupancy, DNase accessibility was assessed in microglia pretreated with GW3965. The addition of LXR agonist had no effect on DNase accessibility at the *Nos2* promoter (Figure 5C).

### LXR activity is reduced in the setting of CNS inflammation

**Figure 6:**
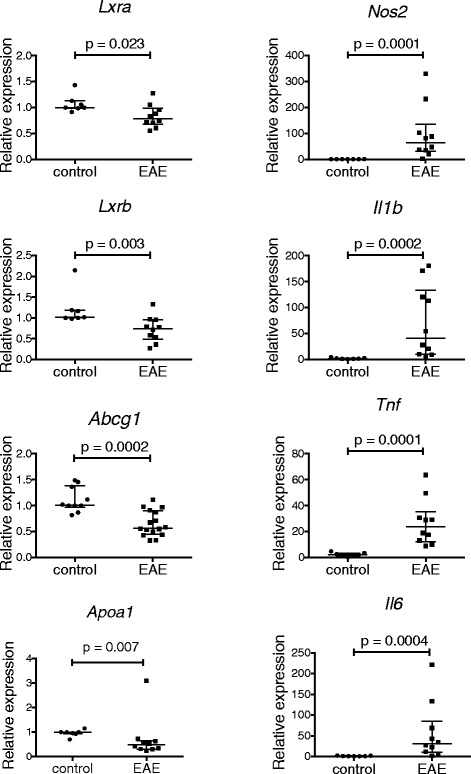
**LXR activity is reduced in the setting of CNS inflammation.** Total RNA was isolated from the spinal cord of experimental allergic encephalomyelitis (EAE) and control mice 12 to 14 days post-induction. RNA levels (relative expression) for *Lxra*, *Lxrb*, *Abcg1*, *Apoa1*, *Nos2*, *Il1b*, *Tnf* and *Il6* were measured by real-time RT-PCR. Control mice received complete Freund’s adjuvant and pertussis toxin without MOG. *Line and error bars* indicate median with interquartile range. Mann–Whitney test of significance.

The inhibitory effect of LXR on microglial *Nos2* indicates a potential role for LXR in modulating CNS inflammation. LXR activity during CNS inflammation, however, has not previously been described. We used EAE as an animal model of CNS inflammation. To assess LXR levels and activity, RNA was isolated from the spinal cords of EAE and control mice at 12 to 14 days post-induction (peak of clinical disease). RNA expressions of *Lxra*, *Lxrb* and the LXR-dependent genes *Abca1*, *Abcg1* and *Apoa1* were determined by real-time RT-PCR. *Lxra*, *Lxrb*, *Abcg1*and *Apoa1* expressions were significantly reduced in EAE compared to control animals, indicating diminished LXR activity in the setting of EAE (Figure 6). *Abca1* levels were not significantly altered (data not shown). *Nos2*, *Il1b, Tnf and Il6* levels were significantly elevated in EAE compared to control animals, indicating the activation of proinflammatory genes during EAE (Figure 6).

### LXR activation is associated with delay in onset of EAE and induction of reverse cholesterol transport genes in the CNS

**Figure 7:**
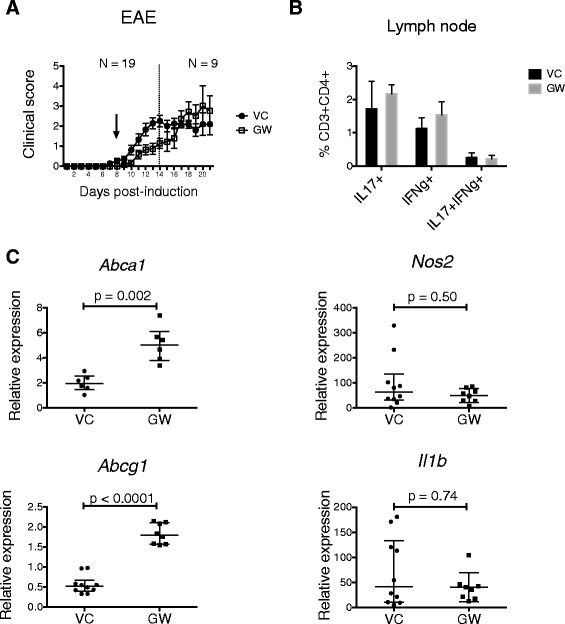
**LXR activation is associated with delay in onset of EAE and induction of reverse cholesterol transport genes in the CNS.** Animals induced to undergo EAE were administered daily intraperitoneal injections of GW3965 (*GW*) or vehicle (*VC*) beginning day 8 post-induction (*arrow*). Animals were killed at day 14 to obtain lymph nodes, spleen and spinal cord. All animals received daily injections up to 14 days post induction (*N* = 19 for the GW group, *N* = 22 for VC group). A subset of animals (*N* = 9 for the GW group, *N* = 12 for the VC group) received daily injections up to 21 days post induction. **A** Clinical score (mean ± SE) of EAE over time for GW and VC treated animals. **B** The frequency interleukin-17A positive (IL17+) and/or interferon-γ positive (IFNg+) CD3 + CD4+ T cells in the lymph nodes (cervical and axillary) of GW and vehicle VC treated animals at day 14 post induction. **C** CNS expression of reverse cholesterol transport genes (*Abca1* and *Abcg1*) and inflammatory genes (*Nos2* and *Il1b*) for VC and GW treated animals. *Line and error bars* indicate median with interquartile range. Mann–Whitney test of significance.

Animals were administered GW3965 during the effector phase of EAE to determine the effect of LXR activation on CNS inflammation. Following active induction of EAE, animals were administered daily intraperitoneal injections of GW3965 (25 to 30 mg/kg) or vehicle (DMSO) beginning at day 8 post-induction. Animals administered GW3965 had significantly reduced cumulative clinical scores compared to vehicle treated animals at day 14 post induction (5.1 ± 1.4 vs. 10.4 ± 6.3, *p* = 0.049), indicating delayed onset of clinical disease in LXR agonist treated animals. Analysis of a subset of animals treated for up to 21 days post induction showed that at later time points the disease severity was comparable between GW3965 and vehicle treated animals (Figure 7A). These results suggest that LXR activation impacts the acute, but not chronic, phase of EAE.

The induction phase of EAE involves peripheral lymphocyte activation, whereas the effector phase of EAE involves the establishment of CNS inflammation. A previous study indicated that LXR influences Th17 lineage differentiation, which is critical to induction of EAE [14]. To assess the effect of LXR activation on peripheral lymphocytes during EAE, intracellular interleukin-17 and interferon-γ were detected in CD4 + T lymphocytes obtained from lymph nodes and spleen to identify Th1 and Th17 cells by flow cytometry. There were no significant differences between LXR and vehicle treated animals with respect to the frequency of Th1 and Th17 cells in the lymph node at day 14 post induction (Figure 7B). Similar results were obtained for splenic T cells (data not shown).

To assess CNS LXR activity and CNS inflammation, RNA was isolated from spinal cords of EAE and vehicle treated animals to measure LXR-dependent gene expression. The LXR-dependent genes *Abca1* and *Abcg1* were significantly increased in GW3965 treated EAE animals compared to vehicle treated animals, indicating increased expression of genes involved in reverse cholesterol transport. RNA expressions of inflammatory genes were also measured. Neither the *Nos2* nor the *Il1b* gene expressions were significantly altered by the administration of LXR agonist GW3965 (Figure 7C). *Tnf* expression was also not significantly altered by LXR agonist administration (data not shown). Thus, the administration of LXR agonist had a more reliable impact on the activation of LXR-dependent genes involved in reverse cholesterol transport than on repression of inflammatory genes, including *Nos2*, in the context of EAE.

## Discussion

Persistent microglial activation has been implicated in the pathogenesis of a number of neuroinflammatory and neurodegenerative disorders [2]. Reactive oxygen species, reactive nitrogen species and inflammatory cytokines have been identified as potentially deleterious products of chronic microglial activation in these disorders, leading to oxidative stress and mitochondrial dysfunction [3]. Mechanisms that inhibit microglial activation, therefore, have the potential to modulate deleterious effects of microglial response in neuroinflammatory and neurodegenerative diseases.

In this report, we confirmed that the activation of the nuclear receptor LXR modifies microglial response to the inflammatory mediator LPS *in vitro*. In particular, LXR activation inhibited microglial production of the reactive nitrogen species nitric oxide. We observed partial inhibition of IL-1β production. LXR-dependent inhibition of nitric oxide and IL-1β production corresponded to reductions in *Nos2* and *Il1b* expressions. The effect of LXR activation on TNF production was less reliable. These results are in agreement with previous studies that identified *Nos2* and *Il1b*, but not *Tnf*, as LXR-repressed genes [25]. The effect of LXR activation on microglial response to interferon-γ was less reliable. There was a trend to decreased nitric oxide production by microglia pretreated with LXR agonist that did not reach statistical significance in the setting of interferon-γ stimulation, possibly indicating a smaller magnitude of LXR-dependent inhibition. Stimulus-specific differences in LXR-dependent inhibition are likely to be important in neuroinflammatory diseases such as multiple sclerosis, where both Toll-like receptor and interferon-γ signaling have been reported [26-29].

LXR activation did not inhibit nuclear translocation of NF-kappaB1 p50, indicating that the mechanism of LXR-dependent repression of inflammatory genes does not involve limiting the amount of NF-kappaB transcription factor available for binding DNA in the nucleus. A previous study showed that ligand-dependent transcriptional repression by LXR involves post-translational modification by SUMOylation and subsequent stabilization of the LXR-NCoR complex on the promoters of target genes [7]. Histone deacetylase (HDAC) and BRG1 are components of the NCoR complex, suggesting that the mechanism of LXR-dependent transcriptional repression involves epigenetic modification [22,30]. We detected peak of LPS-induced NF-kappaB1 p50 binding at −159 to −15 bases upstream of the *Nos2* transcription start site, which corresponds to the previously reported proximal enhancer cluster on the *Nos2* promoter [31]. In addition, we detected histone 4 acetylation upstream of the *Nos2* gene in response to LPS stimulation of microglia, with the peak of histone 4 acetylation detected between −551 to −359 bases upstream of the *Nos2* transcriptional start site. Thus, the peaks of NF-kappaB1 p50 binding and histone 4 acetylation are within 2 to 3 nucleosome distances of each other upstream of the *Nos2* gene. Inhibition of histone acetyltransferase reduced NF-kappaB1 p50 binding on the *Nos2* promoter, suggesting that histone acetylation results in promoter configuration that permits p50 occupancy. Both histone 4 acetylation and p50 binding were partially inhibited by LXR activation, providing additional evidence for a mechanistic link between histone acetylation and NF-kappaB1 p50 binding on the *Nos2* promoter. The addition of HDAC inhibitor or HDAC3 siRNA partially reversed the LXR-dependent repression of nitric oxide production, indicating that LXR-dependent gene repression may be vulnerable to HDAC inhibitors. The interpretation of HDAC inhibitor/siRNA experiments are confounded by that fact that the addition of HDAC inhibitor alone or HDAC3 siRNA alone resulted in increased nitric oxide production or *Nos2* expression above baseline, indicating that HDAC activity exerts basal repression of *Nos2* transcription independent of LXR ligand. DNase accessibility assay indicated that unlike the *Tnf* promoter region, which demonstrated constitutive nucleosome depletion, the *Nos2* promoter appears to be an activation-dependent, inducible nucleosome-depleted region in the microglia. In the resting state, the *Nos2* promoter region showed high nucleosome occupancy. Stimulation with LPS resulted in depletion of nucleosome on the *Nos2* promoter, indicating a shift to euchromatin. Addition of LXR agonist did not alter DNase accessibility at the *Nos2* promoter. Together, these data suggest that the mechanism of LXR-dependent *Nos2* repression involves histone deacetylation that likely alters the local transcription factor access to response elements without a change in the regional nucleosome occupancy.

A key question addressed by this study is whether or not LXR activation can regulate CNS inflammation. A related question is whether or not endogenous LXR activity is a physiologic regulator of CNS inflammation. A previous study reported that LXR knockout animals showed increased clinical disease severity during EAE that was explained by the loss of inhibitory influence on lymphocyte Th17 lineage differentiation [14]. Thus, LXR appears to exert physiologic control over peripheral lymphocyte activation during the induction phase of EAE. In the CNS compartment, however, we found reduced expression of LXR and LXR-dependent genes, indicating that the endogenous LXR activity is, in fact, diminished in the context of CNS inflammation. The reduction in LXR and LXR-dependent genes in the CNS is consistent with previous reports of LXR expression in several other organs. In hepatocytes, renal cells and adipocytes, induction of acute phase response by LPS, TNF and/or IL-1β is associated with reduction in nuclear receptor levels including LXR [32-34]. Thus, inflammatory cytokine-induced suppression of LXR expression also appears to occur in the CNS.

The administration of LXR ligand, nevertheless, partially reversed the diminished LXR activity during EAE. LXR ligand administration increased the expression of LXR-dependent genes involved in reverse cholesterol transport including *Abca1* and *Abcg1* above those in vehicle treated animals, indicating upregulation of LXR activity in the CNS. The administration of LXR ligand had a less reliable effect with respect to repression of inflammatory genes. In particular, we did not see significant repression of *Nos2* expression in the spinal cord of EAE animals following LXR agonist administration. LXR agonist delayed the onset of clinical disease in EAE, but did not impact the severity of clinical disease at later time points, likely reflecting the failure of LXR activation to establish repression of CNS inflammatory genes in EAE. Several explanations are possible. We found a more reliable inhibitory effect of LXR on LPS-induced microglial activation compared to interferon-γ-induced microglial activation. Therefore, CNS inflammatory conditions such as EAE and MS where both Toll-like receptor and interferon-γ signaling are detected may be less responsive to inhibitory action of LXR [26,27]. Alternatively, the threshold for transcriptional activation and transcriptional repression by LXR may be different in the context of CNS inflammation. Whereas genes involved in reverse cholesterol transport are coordinately regulated by LXR transcriptional activation involving exchange of NCoR for NCoA, the antiinflammatory effects of LXR are under SUMOylation-dependent transcriptional repression [35]. There is evidence that the activation and repression functions of LXR can be uncoupled [36]. Although we found no change in the frequency of Th17 cells at day 14 post induction, it remains possible that the delay in onset of EAE clinical disease in LXR agonist treated animals might reflect an inhibitory effect of LXR activation on peripheral lymphocytes.

There are several limitations to the study. Analysis of gene expression was performed on spinal cord homogenates, representing multiple cell types. It is possible that analysis of isolated microglia/macrophage might have detected a statistically significant effect of LXR activation on inflammatory gene expression. However, LXR-dependent repression of inflammatory genes has been reported in multiple cell types including astrocytes [37] and therefore would have been expected to have broader antiinflammatory effects. This study differs from previous studies testing the effect of LXR agonist in EAE with respect to either onset of drug administration or the specific drug administered. Two previous studies examined the effect of LXR agonist administered during the induction phase [14,38], whereas LXR agonist was administered during the effector phase in the current study. A previous study tested a different LXR agonist (T091317) during the effector phase of EAE [38]. T0901317 has turned out to be a rather non-specific LXR agonist, with additional activity toward pregnane X receptor and farnesoid X receptor, not seen in GW3965 [39,40].

## Conclusions

In conclusion, LXR activation inhibits microglial *Nos2* expression and nitric oxide production, in part by histone deacetylation leading to impaired NF-kappaB1 p50 binding on the *Nos2* promoter. Endogenous LXR activity is diminished in the setting of CNS inflammation. Administration of exogenous LXR agonist delays the onset of clinical disease and reverses the diminished LXR activity with respect to genes involved in reverse cholesterol transport, but does not significantly inhibit CNS inflammatory genes in EAE.

## Abbreviations

ChIP: Chromatin immunoprecipitation
CNS: Central nervous system
EAE: Experimental allergic encephalomyelitis
HDAC3: Histone deacetylase 3
IL-1β: Interleukin-1β
LXR: Liver X receptor
NOS2: Nitric oxide synthase 2, inducible
RXR: Retinoid X receptor
TNF: Tumor necrosis factor
